# Rapid Analysis for *Staphylococcus aureus* via Microchip Capillary Electrophoresis

**DOI:** 10.3390/s21041334

**Published:** 2021-02-13

**Authors:** Jin Chen, Yu Sun, Xiaogai Peng, Yi Ni, Fengchao Wang, Xiaoming Dou

**Affiliations:** 1 College of Sciences, Shanghai Institute of Technology, 100 Haiquan Road, Shanghai 201418, China; jinchenxl@sit.edu.cn (J.C.); yusunwei@126.com (Y.S.); pengxiaogai@163.com (X.P.); 2 Institute of Photonics and Bio-Medicine, Graduate School of Science, East China University of Science and Technology, 130 Meilong Road, Shanghai 200237, China; xmdou@ecust.edu.cn; 3 Department of Applied Physics, Graduate School of Engineering, Osaka University, Yamadaoka, Suita-City, Osaka 565-0871, Japan

**Keywords:** *Staphylococcus aureus*, *nuc* gene, microchip capillary electrophoresis, space domain internal standard method

## Abstract

*Staphylococcus aureus* (*S. aureus*) is one of the most common pathogens for nosocomial and community infections, which is closely related to the occurrence of pyogenic and toxic diseases in human beings. In the current study, a lab-built microchip capillary electrophoresis (microchip CE) system was employed for the rapid determination of *S. aureus*, while a simple-to-use space domain internal standard (SDIS) method was carried out for the reliable quantitative analysis. The precision, accuracy, and reliability of SDIS were investigated in detail. Noted that these properties could be elevated in SDIS compared with traditional IS method. Remarkably, the PCR products of *S. aureus* *nuc* gene could be identified and quantitated within 80 s. The theoretical detection limit could achieve a value of 0.066 ng/μL, determined by the using SDIS method. The current work may provide a promising detection strategy for the high-speed and highly efficient analysis of pathogens in the fields of food safety and clinical diagnosis.

## 1. Introduction

*Staphylococcus aureus* (*S. aureus*) is a typical Gram-positive bacteria that is widely distributed in the natural world (e.g., air, water, food, and human body). It is one of the greatest pathogens for nosocomial and community infections [1,2,3,4]. This germ is closely related to the occurrence of pyogenic and toxic diseases in human beings, such as pneumonia, endocarditis, and septicemia [5,6,7]. Moreover, it is noteworthy to mention that *S. aureus* is an important foodborne pathogen, second only to *Escherichia coli* [8,9]. Due to the low requirements for reproduction conditions, day-to-day food is easily contaminated by *S. aureus*. After ingesting contaminated food, these *S. aureus* germs will secrete many serotypes of enterotoxins, which may result in the occurrence of food poisoning. Generally, *S. aureus* ranging between 10^6^~10^8^ cfu/g can produce considerable staphylococcal enterotoxins (such as SEA, SEB, SEC, SED, and SEE), resulting in food poisoning [10,11]. Thus, to realize a rapid, precise, and high-performance analysis is an important and meaningful topic for the fields of food safety and clinical diagnosis.

In early studies, the traditional cultivated and immunological avenues were prevalent for *S. aureus* analysis. However, the intrinsic shortcomings of being time consuming and having poor accuracy may limit the further development of these traditional methods. Recently, because of the remarkable features of noninvasive monitoring and extreme accuracy, real-time polymerase chain reaction (RT-PCR) and gene probe approaches have gained great attraction in the field [12,13]. The specific *mec*, *fem*, and *nuc* genes of *S. aureus* have been employed as the target for analysis [14,15]. However, these gene-based methods are not only complicated, but also costly. Since Jorgenson’s pioneering work in 1991 [16], high-speed capillary electrophoresis (HSCE) has become a widespread tool for PCR products’ analysis. Microchip capillary electrophoresis (microchip CE) is an important branch of HSCE, which features rapid analysis speed, facile operation, and effective cost. It is well acknowledged as a desired strategy for biomolecule analysis. Recently, Zhang et al. described a rapid and sensitive detection of *Escherichia coli* by microchip CE with bacteria-specific aptamers [17]. The analysis time was 135 s and separation channel was 25 mm. Li and his coworkers reported a multiplex PCR strategy with microchip CE for the simultaneous detection of four foodborne pathogenic bacteria (*Vibrio parahemolyticus*, *Salmonella*, *Escherichia coli O157:H7*, and *Shigella*) [18].

As it is known, microchip CE is a typical case of online fluorescence detection with an end-point strategy. Remarkably, it presents an excellent ability for biomolecule identification, which is based on the molecule size. However, the quantitative capacity is still a challenge. Commonly, the time-domain internal standard (TDIS) strategy is used for the quantitative analysis in HSCE. It calculates the content of target sample (TS) through the area ratio of electrophoretic peaks between IS and TS [19], while the electrophoretic peak is a curve in the time domain. As it is known, the sample band is spatially distributed in the separation channel, and its actual content is directly related to the spatial distribution of fluorescence, which is a space-domain signal. According to the protocol of online detection, the space-domain fluorescence signal would be transformed to the time-domain signal (electrophoretic peak). However, this transforming relationship is excluded in the TDIS method. So, the quantitative analysis calculated by common TDIS method is doubtful. To improve this concern, we have illustrated the transformation mechanism from space-domain signal to time-domain signal, and proposed a novel and simple-to-use space-domain IS approach (SDIS) for quantitative analysis in our previous work [20]. However, the application of the proposed SDIS approach for microchip CE was not reported.

In the current study, microchip CE coupled with SDIS method was employed for the rapid analysis of *S. aureus*. The *nuc* gene was used as the target gene. Firstly, the properties of SDIS applied in microchip CE were well examined with various electrophoretic conditions (such as different running cycles, matrix concentration, and separation voltage). Then, electrophoretic experiments of PCR products of *nuc* gene were carried out in a lab-built microchip CE system. SDIS was applied for the quantitative analysis of the target gene. Linearity and the theoretical detection limits were further studied. To our knowledge, there has been no similar work to date. Such work may provide a promising analysis avenue for clinical pathogens.

## 2. Experimental Details

### 2.1. Lab-Built Microchip CE System

**Figure 1:**
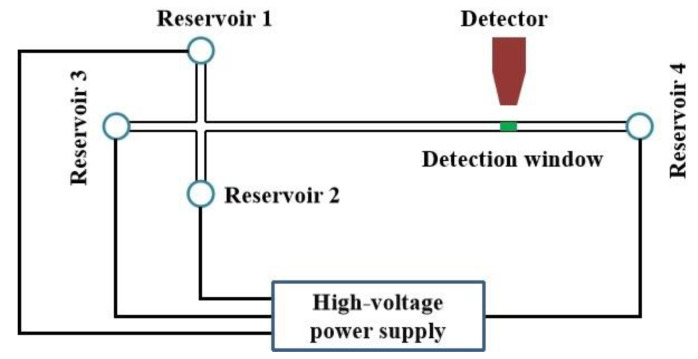
Sketch of the lab-built microchip capillary electrophoresis (CE) system.

In this work, the microchip CE system was designed and built in the lab. As shown in Figure 1, a classical cross-type microchip was employed and fabricated with polydimethylsiloxane (PDMS, SILPOT184, Dow Corning Toray, Japan). The fabrication process of microchip is described in the Supplementary Information. The channel cross-section was 50 μm (width) × 25 μm (depth), and the effective separation length was 20 mm. The distances from the crossing to sample inlet (reservoir 1), sample outlet (reservoir 2), buffer (reservoir 3), and buffer waste (reservoir 4) were 5, 5, 5, and 30 mm, respectively. For sample injection, the voltage value applied to reservoir 2 was 500 V, while that of reservoir 1, 3, and 4 was 0 V (not grounded). In regards to sample separation, the applied voltages on reservoir 1, reservoir 2, and reservoir 3 were 300, 300, and 0 V, respectively, while that of reservoir 4 was varied from 600 V to 1200 V with increments of 200 V.

The optical detection module was mainly equipped on an inverted fluorescent microscope (IX73, Olympus, Japan). The excited light from a mercury lamp (HBO 103W/2, Osram, Germany) was attenuated to 460–495 nm, which well matched the excitation of dye-nucleonic acid conjugate, by an optical filter (U-MWIB3, Olympus, Japan). The emitted fluorescence light was collected by a 100× objective lens and detected by a photomultiplier tube (PMT, H8429-101, Hamamatsu photonics, Hamamatsu, Japan). A data acquisition (DAQ) card and LabVIEW soft (National instrument, Austin, TX, USA) were employed for the raw data collection.

### 2.2. Chemicals and Reagents

Hydroxyethylcellulose (HEC) with a molecule weight of 1300 K was provided by Polyscience (Warrington, PA, USA). 10× Tris-botate-EDTA (TBE) was purchased from Takara of Japan, and diluted to be 0.5× with distilled water. HEC solution was prepared to mass concentrations ranging from 0.4~1%, with above 0.5× TBE for measurements. A 100 bp DNA ladder marker ranging in 100~600 bp was obtained from Ruichu Biotech (Shanghai, China). A standard 400 bp DNA fragment, which was employed for quantitative analysis, was provided by Thermo Fisher Scientific of Waltham, MA, USA. 10^4^× SYBR Green I (SG) from Invitrogen (Carlsbad, CA, USA) was diluted to be a 5× final concentration in HEC solution. Remarkably, SG features a low intrinsic quantum yield (~0.0004) and a high quantum yield of dye-dsDNA complexes (~0.69) compared with other existing dyes. The fluorescence enhancement between free dye and SG/dsDNA complexes is over 1500-fold, which is desirable for the sensitivity in fluorescence detection [21].

### 2.3. PCR Amplification of nuc Gene

In the present work, a SpeedSTAR HS DNA Polymerase Kit form Takara (Kusatsu, Japan) was used for PCR amplification. The *nuc* gene of *S. aureus* was obtained from Synbio Tech (Suzhou, China). Primers of this target gene for PCR process were synthesized by Sangon Biotech (Shanghai, China). Typically, a 30 μL reaction solution was composed of 0.4 μL templates of *nuc* gene, 0.6 μL (100 μM) primers, 0.15 μL DNA polymerase, 3 μL 10× Fast Buffer, 2.4 μL dNTP (2.5 mM), and sterile water. A TP350 thermal cycler (Takara, Japan) was employed for the PCR amplification. For the thermal program, an initial denaturation (95 °C, 2 min) was executed firstly. Then, the PCR thermal-cycling, consisting of (1) denaturation (95 °C, 10 s) and (2) annealing and extension (64 °C, 30 s), was carried out for 30 cycles. According to the designed primers, the expected size of PCR products was 279 bp.

## 3. Results and Discussion

### 3.1. The Protocol of SDIS Method

Regarding fluorescence detection in HSCE, the dye molecules should uniformly intercalate into DNA minor groove to form dye/DNA complexes. In the current work, the SYBR Green I dye that was used has an intercalation interval of around 3~4 bp [22]. This indicates the fluorescence signal (labeled as *FS*) in the space domain is linearly related to analyte content (labeled as *Q*). In capillary, *Q* is determined by sample concentration (labeled as *C*) and injection performance. Thus, according to IS method protocol, there is a relationship as described in Equation (1). Here, *β* is a factor related to injection performance.

(1) $$\frac{Q_{TS}}{Q_{IS}}=\frac{\beta_{TS}\cdot C_{TS}}{\beta_{IS}\cdot C_{IS}}=\frac{FS_{TS}}{FS_{IS}}$$

When the DNA band migrates through the detection window, the detector will constantly transform *FS* into a time-domain-detected signal (labeled as *DS*). *DS* is the area of electrophoretic peak. As discussed in our previous work [20], the intrinsic transforming relationship between *FS* and *DS* can be described as Equation (2). Here, *α* is a linear coefficient related to the detection system performance, *f* is the detected frequency, *T_exp_* is the detector’s exposure time, *L_d_* is the detection window’s width, *L_e_* is the effective separation length, and *T* is the migration time of the DNA band.

(2) $$DS=\alpha \cdot f\cdot T\exp\cdot \frac{L_{d}}{L_{e}}\cdot T\cdot FS$$

Since *α*, *f*, *T_exp_*, *L_d_*, and *L_e_* are constants, there is a relationship as expressed as Equation (3). It is noted that the content relationship between TS and IS samples cannot be determined directly by the *DS_TS_/DS_IS_* ratio, because the migration time *T* is involved. So, the validation of the TDIS method is doubtful.

(3) $$\frac{\beta_{TS}\cdot C_{TS}}{\beta_{IS}\cdot C_{IS}}=\frac{FS_{TS}}{FS_{IS}}=\frac{T_{IS}\cdot DS_{TS}}{T_{TS}\cdot DS_{IS}}$$

For the SDIS method, the TS concentration can be calculated by Equation (4). Note that the ideal value of *β_IS_*/*β_TS_* is 1.0 when there is no injection discrimination.

(4) $$C_{TS}=C_{IS}\cdot \frac{\beta_{IS}}{\beta_{TS}}\cdot \frac{T_{IS}}{T_{TS}}\cdot \frac{DS_{TS}}{DS_{IS}}$$

### 3.2. SDIS Performances in Microchip CE

To investigate the SDIS properties in the application of microchip CE, a standard 100 bp DNA ladder marker ranging in 100~600 bp was employed in current study. Microchip CE experiments were carried out under different conditions. The fluorescence signal was detected and recorded to produce electrophoretograms. Photobleaching could affect fluorescence intensity, which may be a possible error factor for quantitative analysis. In our experiment, the exposure time of sample bands passing through the detection window was much smaller than the decay lifetime of SG/dsDNA complexes [23]. So, the photobleaching could be neglected in current study.

**Figure 2:**
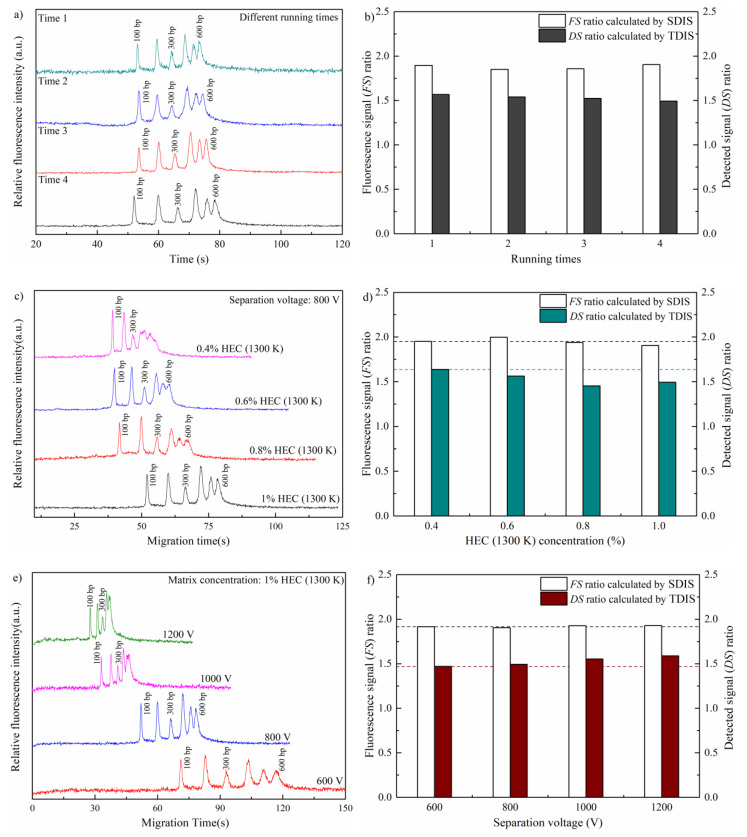
Electrophoretograms of the standard 100 bp ladder marker ranging between 100~600 bp: (**a**) run-to-run tests performed in 1% hydroxyethylcellulose (HEC) (1300 K) at 800 V; (**b**) fluorescence signal (*FS*) and time-domain-detected signal (*DS*) ratios of run-to-run tests; (**c**) HEC (1300 K) matrix concentration ranging 0.4–1%; (**d**) *FS* and *DS* ratios of matrix concentrations ranging 0.4–1%; (**e**) separation voltages ranging 600–1200 V; (**f**) *FS* and *DS* ratios of separation voltages ranging 600–1200 V. The dash lines in (**d**) and (**f**) were the auxiliary lines for observation.

Figure 2a shows the electrophoretograms of run-to-run tests performed four times in 1% HEC (1300 K) at 800 V. In the current study, a 300 bp DNA fragment of the standard marker was set as the IS sample, while a 100 bp fragment was considered as the TS sample. It was found that the standard deviation (*STD*) values for the migration time of 100 and 300 bp were calculated as 0.75 and 0.96 s separately. The coefficient of variation (*CV*) values were 1.42% and 1.48%, respectively. This suggested the separation based on the lab-built microchip CE system showed a stable repetition property in migration time, which was favorable for the qualitative analysis. Figure 2b shows *FS* ratio and *DS* ratio calculated by SDIS and TDIS, respectively. As observed, the *STD* and *CV* values of 100/300 bp *DS* ratio were 0.031% and 2.01%, respectively, while those of *FS* ratio were 0.027% and 1.44%. It suggested no obvious variation of precision between TDIS and SDIS. This can be expected due to the stable repetition property shown in Figure 2a. However, a considerable difference between *FS* ratio and *DS* ratio was observed in Figure 2b. As explained in Section 3.1, it was the spatial distribution of fluorescence that directly related to the amount of sample band, rather than the time-dependent signal in electrophoretograms. Peak area in electrophoretograms could not directly reflect the real amount of sample band. In SDIS, by investigating the detection process of electrophoresis, it was found that a correction factor must be considered for IS method as shown in Equation (4). This was why the *FS* ratio was different from the *DS* ratio, which suggested an improvement in accuracy. This was also confirmed by our previous work [20]. In the case of Figure 2b, the average *DS* ratio was 0.345 smaller than the *FS* ratio. It meant there was an 18.4% underestimation of the peak area ratio, and then the amount of target fragment, by using TDIS.

The separations under different electrophoretic conditions (including matrix concentration and separation voltage) were carried out to examine the reliability of present SDIS method in microchip CE application. Figure 2c displays the electrophoretograms undertaken by different HEC (1300 K) matrix concentrations, varied in 0.4~1% with increments of 0.2%, when the separation voltage was a constant of 800 V. Figure 2e describes the separation diagrams of 600~1200 V separation voltages changed with an interval of 200 V, when the HEC (1300 K) matrix was in 1% concentration. As seen, the DNA band showed different peak shape or area as the electrophoretic conditions varied. Figure 2d,f shows *FS* and *DS* ratios of 100/300 bp in various electrophoretic conditions. Compared with the obvious variation of peak shape or area in electrophoretograms, the changes of *FS* and *DS* ratios were slighter. Regarding the matrix concentration tests, the *STD* and *CV* values of *DS* ratio were 0.081 and 5.26%, respectively, while those of *FS* ratio were 0.038 and 1.97%. For the separation voltage experiments, the *STD* and *CV* values of *DS* ratio were 0.054 and 3.56%, separately, while those of *FS* ratio were 0.011 and 0.55%. In both situations, the *STD* and *CV* values of *FS* ratio were a little smaller than those of *DS* ratio. This suggested a further improvement of reliability in SDIS. In addition, a considerable difference between *FS* ratio and *DS* ratio also appeared. As discussed above, this indicated an accuracy improvement. It was noteworthy that *DS* ratio in Figure 2d gradually decreased, while matrix concentration rose from 0.4% to 0.8%. The reason was implied in Equation (3). For a given pair of TS and IS samples with concentration *C_TS_* and *C_IS_*, respectively, and assuming the injection condition does not change, the *β_TS_C_TS_*/*β_IS_C_IS_* value in Equation (3) is constant. If migration time ratio *T_TS_*/*T_IS_* decreases (i.e., *T_IS_*/*T_TS_* increases), *DS* ratio must decrease. If migration time ratio *T_TS_*/*T_IS_* increases (i.e., *T_IS_*/*T_TS_* decreases), *DS* ratio must increase. We measured the migration time of 100 bp (TS) and 300 bp (IS) in Figure 2c, and calculated their ratios. The values of migration time ratio *T_TS_*/*T_IS_* with matrix concentration rising from 0.4% to 0.8% were 0.84, 0.78, and 0.75, respectively. The descending trend of migration time ratio *T_TS_*/*T_IS_* was in good accordance with that of *DS* ratio observed in Figure 2d. The situation of matrix concentration 1.0%, which seemed to be against the trend, also conformed to the regulation. Due to this, the value of *T_TS_*/*T_IS_* with concentration 1.0% was measured to be 0.78. It was a little bigger than that of concentration 0.8%, which was consist with the bigger *DS* ratio of concentration 1.0% shown in Figure 2d. Similarly, the values of migration time ratio in Figure 2e with separation voltage ranging from 600 V to 1200 V were 0.77, 0.78, 0.81, and 0.83, respectively. The ascending trend of migration time ratio was in accordance with that of *DS* ratio in Figure 2f. It should be pointed out, though the *DS* ratio in our experiments (Figure 2d,f) presented dependence on matrix concentration and separation field, it was the surface phenomenon. The variation of *DS* ratio was directly determined by the migration time ratio, and the variation of migration time ratio might have been induced by matrix concentration, separation field, or other experiment conditions, with monotonic or nonmonotonic regulation, or even by instable experiment performance. This meant the traditional TDIS method, which does not take migration time into consideration, could not resist the error due to the variation of migration time derived from experiment conditions. Contrarily, the proposed SDIS method dealt with this problem by an intrinsic correction factor (*T_IS_*/*T_TS_*) in Equation (4). Thus, the effect of changing migration time was compensated. This was why the *DS* ratio in all the situations (Figure 2b,d,f) only presented small fluctuations no matter how the experiment conditions changed.

### 3.3. Analyses of S. aureus Based on Microchip CE System Combined with SDIS Approach

**Figure 3:**
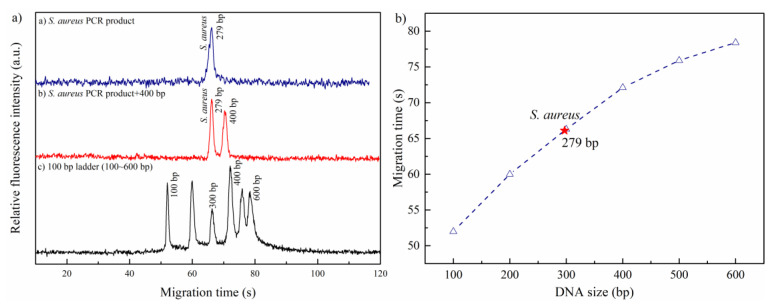
(**a**) Identification for the PCR products of *nuc* gene; (**b**) the plot of DNA size versus migration time (the red star represents the PCR product of *S. aureus*). The electrophoresis was performed in 1% HEC (1300 K) at 800 V.

In this study, the standard 100 bp DNA ladder marker, including six bands ranging from 100 to 600 bp, was employed for the identification of target PCR products. The analysis was performed in 1% HEC (1300 K) at 800 V. According to PCR protocol, the size of PCR products amplified by the designed primers was 279 bp. As seen in Figure 3a, the DNA fragments in the 100~400 bp range almost featured a baseline separation, which was favorable for the determination of target sample. Figure 3b plots the calibration curve of fragment size versus migration time based on standard 100 bp DNA ladder marker (100~600 bp). Note that the PCR products of *nuc* gene had a good location, which was in good accordance with this calibration curve. This indicated a good identification capacity for the target sample in the lab-built microchip CE system. In addition, the PCR sample with the 400 bp IS sample could be well and rapidly identified within 80 s. This was desirable for highly efficient analysis in clinical applications. 

**Figure 4:**
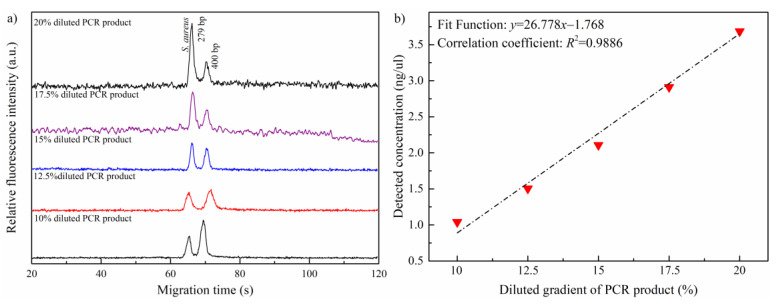
(**a**) Electrophoretograms for PCR products of *nuc* gene in different concentrations; (**b**) the relationship between the detected concentration and the diluted concentration of the PCR products. The electrophoretic conditions were the same as that of in Figure 3.

Regarding the quantitative analysis, the obtained PCR products were diluted to a desired concentration gradient of 10%, 12.5%, 15%, 17.5%, and 20% to investigate the real application performance of proposed SDIS method in microchip CE. Noted that the standard 400 bp DNA fragment was employed as IS sample with a concentration of 2 ng/μL. The electrophoretic conditions were the same as those of the identification process. Figure 4a shows the electrophoretograms of the PCR samples in different concentrations with IS sample. As seen, the detected peak intensity featured a regular decreasing trend as the concentration declined. Figure 4b displays the detected concentration for diluted PCR sample, which was calculated by Equation (4) of the SDIS method. A detailed description of the data in Figure 4b is summarized in Table 1. As observed, the detected concentration of PCR product was linearly related to its diluted concentration, where the regression function was *y* = 26.778*x* − 1.768 and the correlation coefficient *R*^2^ was 0.9886. Furthermore, the theoretical detection limit for the PCR products of *nuc* gene was 0.066 ng/μL, which was determined by the above regression function. 

## 4. Conclusions

In the current study, a lab-built microchip CE system coupled with SDIS method was employed for the rapid identification and quantification analysis of *S. aureus*. The *nuc* gene was used as target gene for analysis. The application performances (including precision, accuracy, and reliability) of the proposed SDIS method in microchip CE were well investigated by using a standard 100 bp DNA ladder marker ranging in 100~600 bp. The run-to run tests and the electrophoretic experiments in different matrix concentrations and separation fields were carried out. Analyses of *STD* and *CV* of *FS* and *DS* ratios suggested the precision and reliability were slightly improved by SDIS. Moreover, there was a considerable difference between *FS* ratio and *DS* ratio, which indicated an elevation of accuracy by SDIS. An error over 18% was corrected in the present work. In addition, it was found that the results of traditional TDIS were directly determined by migration time ratio of TS and IS, which were disturbed by experimental conditions. This problem was also dissolved by the proposed SDIS method. Then, the PCR products of *nuc* gene were analyzed by the lab-built microchip CE. The target fragment could be identified and quantified within 80 s. The linearity was studied by a series of samples with different concentrations. The detected sample concentration calculated by SDIS featured a good linearity with a correlation coefficient *R*^2^ of 0.9886. In addition, the theoretical detection limit was 0.066 ng/μL. The strategy of microchip CE combined with SDIS is favorable for high-speed and highly efficient analysis of pathogens in the fields of food safety and clinical diagnosis.

## Figures and Tables

**Table 1:** Detected concentrations of gradient PCR products of the *nuc* gene.

Diluted Gradient of PCR Product	Repetition Times (N)	Detected Concentration (Mean ± STD)	CV (%)
10%	4	1.039 ± 0.018 ng/μL	1.73%
12.5%	4	1.504 ± 0.043 ng/μL	2.86%
15%	4	2.107 ± 0.028 ng/μL	1.33%
17.5%	4	2.911 ± 0.083 ng/μL	2.85%
20%	4	3.683 ± 0.080 ng/μL	2.17%

## Data Availability

Not applicable.

## Supplementary Materials

The following are available online at https://www.mdpi.com/1424-8220/21/4/1334/s1.
