# Supplementary material for: Rapid Analysis for Staphylococcus aureus via Microchip Capillary Electrophoresis
Source: Sensors (Basel). 2021 Feb 13;21(4):1334. doi: 10.3390/s21041334 (PMC7917630; doi:10.3390/s21041334)
Supplement: Supplementary file 1 [file sensors-21-01334-s001.pdf]

Supporting information

1. Fabrication of microchip

1.1. Preparation of microchip template

Firstly, Su-8 photoresist (GM1060, Resemi, Suzhou, China) was spin-coated onto the silicon substrate with 1335 rpm/s for 40 s. To improve the uniformity, the deposited substrate was relaxed for 10 min. Then, pre-baking was performed with two steps of (65 ℃, 10 min) and (95 ℃, 30 min). Secondly, the deposited layer was exposed with designed mask by lithography machine (ABM/6/350/NUV/DCCD/SA, ABM, USA). The total exposure dose was 550 mJ/cm^2 while the i-line was 365 nm with an intensity of 20 mW/cm^2. Afterward, post-baking was carried out with the same thermal steps as pre-baking. Thirdly, the sample was developed with PGMEA (propylene glycol monomethyl ether acetate) which was purchased from Resemi of Suzhou (China). The developing time was 2.5 min. Finally, the sample was cleaned by isopropanol to obtain the expected microchip template.

1.2. Fabrication of microchip

Typically, un-cured PDMS solution was placed onto the surface of microchip template which was fixed by a lab-built holder. Here, elastomeric base and curing agent were in a ratio of 10:1. Then, the holder was thermal processed at 85 ℃ for 1 h, which would accelerate the curing process. Finally, the cured PDMS sample was stripped from the template, and boned with glass substrate by air plasma process to obtain the using microchip for electrophoresis.
